# Simultaneous medullary and papillary thyroid cancer: two case reports

**DOI:** 10.1186/1752-1947-1-133

**Published:** 2007-11-12

**Authors:** Gianlorenzo Dionigi, Patrizia Castano, Valentina Bertolini, Luigi Boni, Francesca Rovera, Maria Laura Tanda, Carlo Capella, Luigi Bartalena, Renzo Dionigi

**Affiliations:** 1 Department of Surgical Sciences, University of Insubria, Varese, Italy; 2 Department of Human Morphology, University of Insubria, Varese, Italy; 3 Department of Clinical Medicine, Division of Endocrinology, University of Insubria, Varese, Italy

## Abstract

**Background:**

Papillary thyroid carcinoma (PTC) and medullary thyroid carcinoma (MTC) have always been considered different from each other; in their incidence, their cell origin and their histopathological features.

**Case presentation:**

This paper describes two rare cases of the simultaneous occurrence of MTC and PTC in the thyroid gland. Case 1 is unique for different reasons: (a) the patient was affected by both multicentric MTC and PTC; (b) a "composite thyroid carcinoma" with mixed feautures of MTC and PTC carcinomas was found in the istmus of the gland; and (c) these tumors were associated with diffuse lymphocytic-type thyroiditis (LT). Case 2 is notable for the long follow up: 16 years disease free.

**Conclusion:**

There are only 16 reports in the English medical literature describing a total of 20 cases of concurrent occurrence of both PTC and MTC in the same thyroid gland. We discuss whether the finding of another cancer in these patients was coincidental or from possible activation of a common tumorigenic pathway for both follicular and parafollicular thyroid cells.

## Background

Papillary thyroid carcinoma (PTC) is the most common histological type of thyroid cancer (75–80%) [1]. It derives from the follicular cells of the endoderm. [1]. PTC produce thyroglobulins and thyroid hormones [1]. Medullary thyroid cancer (MTC) represents only 5–8% of cases [2]. MTC has a different embryological origin: it derives from parafollicular cells of the ultimobranchial body of the neural crest. MTC secrets calcitonin and other hormonal peptides and is considered part of the amine precursor uptake and decarboxilation system of the thyroid [2]. MTC may occur either as a hereditary or a nonhereditary entity. Hereditary MTC can occur either alone as familial MTC (FMTC) or as the thyroid manifestation of multiple endocrine neoplasia type 2 syndromes [2].

Thyroid carcinoma is frequently associated with genetic alterations. Activating point mutations of RET proto-oncogene have been demonstrated to be causative of the familial form of medullary thyroid cancer, both as isolated FMTC and associated to MEN 2A and 2B [2]. Somatic rearrangements of RET designated as RET/PTC (from papillary thyroid carcinoma) were identified in papillary thyroid carcinoma before RET was recognized as the susceptibility gene for MEN2. There are now at least at least 15 types of RET/PTC rearrangements involving RET and 10 different genes [1].

PTC is characterized by the presence of papillae and specific cellular changes such as epithelial cells situated on basal membranes covering stromal fibres and thin capillaries, round laminated calcifications (psammoma bodies), ground-glass nuclei and cytoplasmatic pseudoinclusions [1]. MTC is composed of solid nests and infiltrating formations of polygonal or spindle-shaped cells, oxyphilic small cells and, sometimes, anaplastic features. The presence of amyloidal deposits and positive immunohistochemistry usually confirms the diagnosis of MTC [2].

Patients with PTC have the highest 10-year relative survival (0.98). Prognosis of MTC is generally worse than PTC (0.80) [1,2]. PTC happens to be a multicentric tumor and tends to spread to the regional lymph nodes in the early stage of the disease [1]. In fact, PTC is associated with cervical lymph node metastases in 30% to 90% of patients [1]. FMTC is often multifocal and bilateral [2]. The incidence of positive lymph nodes correlates with the size of the primary lesion at the time of diagnosis: 60% of patients have positive lymph nodes if the MTC is larger than 2 cm [2].

PTC and MTC have always been considered different one from each other in terms of their incidence, their cell origin and their histopathological features. The concurrent occurrence of both in the same patient is rare, in fact there are only 16 reports describing a total of 20 cases in the English language medical literature [3,4].

The aim of this report is to describe two additional cases of this rare association. Unique pathological figures are presented.

## Case 1

**Figure 1:**
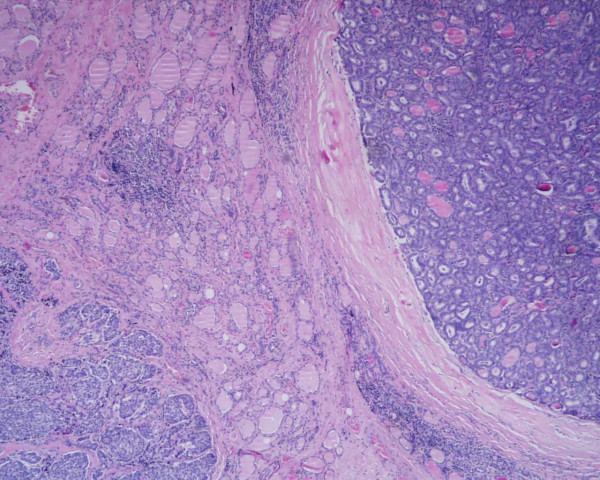
Coexistence of (right) medullary and (left) papillary carcinoma with follicular aspect (H&E, 100×).

A 65-year-old man was referred to our Department with a right cervical mass on April 2005. There was no apparent family history of endocrine disorders. The patient had not undergo any previous external radiation therapy. Blood pressure was normal. His serum levels of calcium, thyroid stimulating hormone and free thyroxine were normal. The baseline serum calcitonin level was 294 pg/ml (normal < 100 pg/ml). There were no antithyroid autoantibodies. On physical examination a rough nodule, about 4 cm in size, was palpated on the right side of the neck. The ultrasonography (US) showed a heterogeneous nodule, 4 cm in size, in the right lobe of the thyroid and multiple nodules with accompanying calcifications in the left lobe. A single fine-needle aspiration biopsy of the right lobe nodule revealed giant cells with enlarged nuclei and metachromatic cytoplasmic granules. Abdominal US was negative for adrenal nodules as well as urinary catecholamines and metanephrine levels were within normal limits. Total thyroidectomy plus central compartment lymph node neck dissection were performed. Macroscopically the right thyroid lobe measured 7 × 4 × 3.5 cm, left lobe of the thyroid measured 4.3 × 2.5 × 2 cm. There was a right solitary white well-circumscribed nodule of 4 cm in size, associated with a small nodule of 0.4 in the isthmus and a nodule (diameter 0.5 cm) with fine calcification in the left lobe. Microscopic examination of the right nodule confirmed medullary carcinoma of the thyroid with neoplastic spindle-shaped cells forming nests with pale, abundant granular cytoplasm. Immunohistochemical stain was positive for calcitonin and synaptophysin, and was negative for thyroglobulin and p53. Microscopic examination of the nodule (0.4 cm) of the isthmus revealed co-existence of medullary and papillary carcinomas with follicular aspect (Figure 1). Microscopic appearance of the left lobe thyroid nodule revealed papillary carcinoma with branching papillary structure lined by cells with empty appearing nuclei and characteristic nuclear pseudoinclusions. Finally most of thyroid tissue was accompanied by diffuse lymphocyte-type thyroiditis (LT). 4 our of 19 lymph nodes of the central compartment were positive for metastasis of medullary thyroid cancer. TNM for PTC was pT2, N0 and for MTC was stage 3. The patient was discharged, without complications, on the third postoperative day. The patient underwent radioiodine treatment. Analysis of tumor tissue for the RET oncogene mutations was negative. Six months postoperative serum calcitonin and TG levels were within normal range.

## Case 2

**Figure 2:**
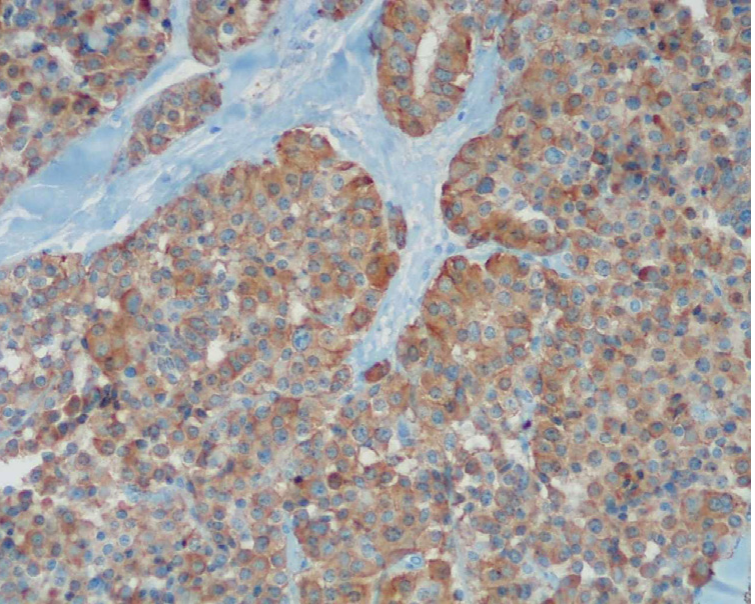
Positive immunostaining reaction for CGRP and calcitonin (DAB-Haematoxylin 400×).

**Figure 3:**
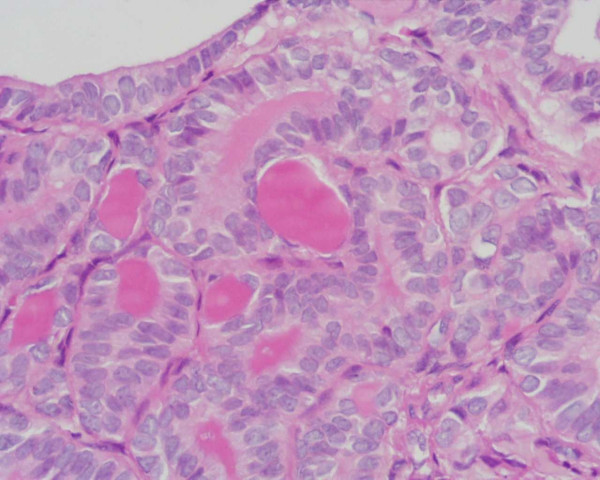
Papillary thyroid carcinoma: the neoplastic cells show overlapping clear nuclei and abundant eosinophilic cytoplasm (tall cell variant) (H&E 400×).

In May 1990 a 34-year-old woman, with no relevant past or family history, underwent a surgical biopsy of enlarged lymph nodes on the left side of the neck. Histological result was compatible with metastasis of carcinoma, possibly originated from her thyroid. Subsequent US of the gland showed an ipo-echoic irregular nodule in the left lobe, with some micro calcifications, of 2.7 × 1.9 cm in size and another little nodule with similar features near the isthmus. A small ipo-echoic nodule of 1 cm in size, probably a lymph node, was described on the left side of trachea. No pathological features were noted in the right lobe. The scintigraphy of the thyroid showed a cold area corresponding to the nodule described on the left lobe at US. Her serum level of thyroid hormones was normal, while calcitonin was >1000 pg/ml and CEA was 661 pg/ml. Total thyroidectomy and left neck dissection were performed. Histological result showed a solid white nodule in the thyroid lobe (2 cm in size) consistent with medullary thyroid carcinoma with solid pattern, nests of spindle-shaped cells and amyloidal deposits, positive reaction for CGRP and calcitonin (Figure 2). Another grey-white nodule with calcifications (0.3 cm in size) was described in the right lobe, consistent with papillary thyroid carcinoma, with neoplastic cells showing overlapping clear nuclei and abundant eosinophilic cytoplasm (tall cell variant) (Figure 3) (tumor categories were pT2, N+). The patient was discharged without complications and surgical treatment was followed by irradiation of the neck (46 GY of total dose). During follow up calcitonin levels were between 300 and 200 pg/ml. A new scintigraphy with ^131^I and MIBG was performed on September 1990: it demonstrated a residual neoplasm in the cervical area. Another surgical excision biopsy was executed and 5 lymph nodes were removed. Histological result showed metastasis of papillary carcinoma. No more therapy was carried out. The patient is still alive, free from illness (now 16 years disease free).

## Discussion

MTC is a rare and aggressive type of thyroid cancer with several distinctive features that distinguish its management from PTC. Since MTC was first recognised as a distinct tumour in 1959, it became clear that MTC is more difficult to cure than PTC and has higher rates of recurrence and mortality [1,2]. In addition, unlike PTC cancer, there is no known effective systemic therapy for MTC [2].

These cases are peculiar for the unique pathological features. In particular, case 1 is extremely rare and of high interest for different reasons: (a) the patient was affected by both *multicentric *MTC and PTC (this is the first report of a multifocal occurrence of different histological types of thyroid carcinomas in the same gland); (b) in addition a *composite thyroid carcinoma *with mixed features of MTC and PTC carcinomas was found in the isthmus of the gland; (c) the association of these tumours with diffuse *lymphocyte-type thyroiditis*. Case 2 is notable for the long follow up (16 years disease free).

The question is whether the finding of another thyroid cancer in these patients was coincidental or from possible activation of a common tumorigenic pathway for both follicular and parafollicular thyroid cells. It is apparent that in the last decade, carcinoma of the thyroid is becoming increasingly prevalent. In fact, the incidence of thyroid carcinoma in Europe has increased during the period 1978–1997, by 3% per year, largely due to an increase rate of PTC [1]. In Italy, PTC is the most frequent thyroid cancer and shows a significant increase of incidence rates [1].

The pathogenesis of thyroid malignancy is unknown, but an underlying common genetic drive has been hypothesized. The process of thyroid oncogenesis is conceived to be a series of events induced by genetic and environmental factors which alter thyroid cell division and growth control. These factors can be considered as initiators and promoters [5]. The first class of factors induce incipient tumor genesis while the second augments TSH secretion and radically increases tumor growth [5]. Normally silent, intracellular proto-oncogenes can become activated by chromosomal translocations, deletions or mutations and can transform normal thyroid cell into a condition of uncontrolled division and growth [5]. The most significant known cause of thyroid carcinomas is exposure to external or internal ionizing radiation [5]. Several early events have been implicated in the neoplastic transformation of thyrocytes, and recent reports have described the involvement of specific genetic alterations in different types of thyroid neoplasms [5]. Recent studies revealed that the RET proto-oncogene is involved in the oncogenesis of MTC and PTC by activation of its tyrosine kinase either by point mutation or rearrangement [5]. Activating germ-line point mutations in the RET receptor are responsible for multiple endocrine neoplasia type 2-associated MTC, whereas somatic RET rearrangements are prevalent in PTC [5].

In the English language medical literature there are 12 reports describing cases of mixed medullary-papillary carcinomas [3,4]. Apel first proposed the term of "composite thyroid carcinoma" [6]. This can be explained if we consider the theory of a common stem cell, proposed by Ljunberg [7]. According to this theory, the ultimobranchial body is the most likely source of this putative common stem cell because the nests of these cells in the thyroid gland show immunoreactivity for both thyroglobulin and calcitonin, suggesting that the ultimobranchial body have contributed to both the parafollicular and follicular cells [6,7]. In contrast to this theory, Volante proposed that the components of the two carcinomas are not derived from a single progenitor cell, because they observed different patterns of RET proto-oncogene mutation, loss of heterozygosis and X-chromosomal inactivation in a lot of these tumours [8]. Furthermore, it has been supposed that unknown factors are necessary to be present for the stimulation of follicular cells, as well as the detection of these substances in the MTC of mixed medullary and follicular carcinomas and their absence in classical medullary thyroid cells [8]. Recently Rossi reported three cases of MTC/PTC collision tumour in which two mutations, in the RET and BRAF genes, were identified, thus documenting the different genetic origin of these two coexisting carcinomas [9].

Finally, PTC is often accompanied by LT a phenomenon that may give rise to analyses both to pathogenetic mechanisms and to prognostic implications [10]. Coexisting LT has been shown to be associate with lower pT stages [10]. Authors have shown that apart from age (45 years or more), vascular invasion, and lymph node metastases, the absence of LT represents an independent prognostic indicator both for relapse-free and overall survival [10]. Recently, Tamini reported that the prevalence of lymphocytic infiltrate is significantly higher in patients with papillary carcinoma (58%) than in patients with follicular carcinoma (20%) or follicular adenoma (14%): the possibility that an immunologic mechanism involved in the pathogenesis of papillary carcinoma stimulates lymphocytic infiltration in the thyroid tissue through an autoimmune mechanism was suggested [10]. There are no data in literature of the association of MTC and LT.

## Competing interests

The author(s) declare that they have no competing interests.

## Authors' contributions

GD: acquisition of data, PC, LB: study conception and design, VB, FR, RD: analysis and interpretation of data, MLT, LB: drafting of manuscript, CC, RD, LB: Critical revision and supervision
